# Changes in pediatric injuries sustained while engaged in activities where helmet usage is recommended during the COVID-19 pandemic

**DOI:** 10.1186/s40621-023-00449-2

**Published:** 2023-08-01

**Authors:** Brent M. Troy, Kiesha Fraser Doh, Allison F. Linden, Yijin Xiang, Scott Gillespie, Maneesha Agarwal

**Affiliations:** 1 Division of Emergency Medicine/Children’s Healthcare of Atlanta, Department of Pediatrics, Emory University School of Medicine, 1547 Clifton Road NE, 2nd Floor, Atlanta, GA 30322 USA; 2 Division of Pediatric Surgery/Children’s Healthcare of Atlanta, Department of Surgery, Emory University School of Medicine, Atlanta, GA USA; 3 Pediatric Biostatistics Core, Children’s Healthcare of Atlanta/Emory University, Atlanta, GA USA

**Keywords:** COVID-19, Helmet, Bicycle, ATV, Pediatrics

## Abstract

**Background:**

Unintentional injuries, including traumatic brain injuries (TBI), are the leading cause of pediatric morbidity and mortality in the USA. Helmet usage can reduce TBI incidence and severity; however, the epidemiology of pediatric TBI and helmet use is ever evolving. With lifestyle changes potentially accelerated by the pandemic, we predicted a decrease in helmet utilization with an associated increase in TBI during the pandemic compared to the pre-pandemic period.

**Results:**

There were 1093 patients that presented with AWHUR injuries from 2018 to 2020 with an annual increase from 263 patients in 2018 up to 492 in 2020. The most frequently implicated mechanisms included bicycles (35.9%), ATVs (20.3%), skateboards (11.6%), scooters (8.3%), and dirt bikes (7.4%). Unhelmeted patients increased from 111 (58.7%) in 2018 to 258 (64.8%) in 2020. There was not a significant difference in the proportion of injuries that were unhelmeted from 38.9% in 2018–2019 to 35.2% in 2020 (*p* = 0.30), as well as the proportion of head injuries from 2018 to 2019 (24.3%) to 2020 (29.3%) (*p* = 0.07). A significant increase was seen in neurosurgical consultation from 17 (6.5%) in 2018 to 87 (17.7%) in 2020 (*p* = 0.02). Notably, there was an increase in the percentage of publicly insured patients presenting with injuries from AWHUR during 2020 (*p* < 0.001); this group also had suboptimal helmet usage.

**Conclusion:**

This study found an increase in patients presenting with injuries sustained while engaged in AWHUR in relation to the COVID-19 pandemic. Concerningly, there was a trend toward decreased helmet utilization and increased injury severity markers. Further analysis is needed into the communities impacted the most by AWHUR injuries.

## Background

Unintentional injuries are a leading cause of pediatric morbidity and mortality in the USA (CDC 2013). This includes traumatic brain injuries (TBI), a common outcome that contributes to both significant morbidity and mortality. In 2014, there were > 830,000 emergency department (ED) visits and hospitalizations, and > 2500 deaths in children due to TBI (Peterson et al. 2014). Many of these are attributable to recreational activities where helmet usage is recommended (AWHUR) including bicycling, scootering, riding all-terrain vehicles (ATVs), snow-sports (skiing, snowboarding), skating (ice, roller, blading), skateboarding, and participation in equestrian sports. Combined, these activities have resulted in over 42,000 pediatric ED visits annually for nonfatal TBI, with bicycle injuries causing the highest incidence of TBI (CDC 2013; Graves et al. 2013).

Participation in AWHUR is important for pediatric well-being, but can result in preventable injuries, especially if helmets are not used. Multiple studies have established the efficacy of helmets in reducing the incidence and severity of TBI in these activities (Denning et al. 2014; Buehler and Pucher 2021; Bowman et al. 2009; Merrigan et al. 2011; Thompson et al. 1996; Fischer et al. 2012; Sadeghian et al. 2017; Short et al. 2018; Fenerty et al. 2013; McGeehan et al. 2004; Donohue and Miller 2020). The American Academy of Pediatrics’ (AAP) recently published a policy statement reaffirming the importance of proper helmet use during common childhood activities; the Centers for Disease Control and Prevention (CDC) makes similar recommendations (CDC 2011; Lee et al. 2022). Interventions have been shown to increase helmet use in children, including community-based helmet campaigns, which have been shown to be effective in reducing brain injuries through helmet usage (Hooshmand et al. 2014; Kirsch and Pullen 2003; Lachapelle et al. 2013; Wesson et al. 2000; Cripton et al. 2014). However, these efforts can be costly. Identification of populations where helmet use is most problematic could help target education initiatives (Selassie et al. 2004; Tepas et al. 2011).

Pediatric participation in AWHUR may have changed during the COVID-19 pandemic. Although data are limited, there were reports of increased bicycle use during the initial months of the pandemic (Denning et al. 2014 Dec). While state-to-state public health approach to the pandemic varied greatly, on March 14th, 2020, the state of Georgia was placed into a Public Health State of Emergency due to the pandemic. Closure of schools and non-essential venues during the pandemic resulted in a significant change in the lifestyle of families and their children through necessary stay-at-home orders. Children were away from school and given more time for solo outdoor activities, such as bicycling (Donohue and Miller 2020). Additionally, adult supervision may have been altered as families adjusted to the necessary societal changes to mitigate the impact of COVID-19.

Our primary aim was to determine the incidence of injuries and helmet usage involving AWHUR before and during the COVID-19 pandemic. We additionally aimed to characterize these changes via mechanism, characteristics, and severity of injury before and during the pandemic. We hypothesized that there has been an increase in pediatric injuries from AWHUR and decrease in helmet use during the pandemic compared to the same months in previous years prior to the pandemic.

## Results

### AWHUR injury demographics

A total of 1093 patients presented with injuries from AWHUR over the time frame (Table 1). The majority of patients (85%) were 5–14 years old. Approximately, 66% of the patients were male. Most patients identified as white (67.5%) or black/African American (23.7%); 11.9% of the patients were of Hispanic or Latino ethnicity. Overall, 46.8% of patients had public insurance, but the proportion of patients with public insurance increased from 40 to 51% (*p* = 0.006) with a gross increase from 104 patients in 2018 up to 252 in 2020. AWHUR injuries grossly increased annually from 263 patients in 2018 to 492 in 2020. Additionally, there were 1.85 injuries per 10,000 children in 2018 up to 2.38 injuries per 10,000 in 2019 and up to 3.43 injuries per 10,000 in 2020, with 1.37 [95% CI 1.27, 1.48] change rate per 10,000 per year. (*p* < 0.001).

**Table 1:** AWHUR patient demographics

	Overall	2018	2019	2020	*p*	Covid	Pre-Covid	*p*
	1093	263	338	492		492	601	
*Age (%)*								
< = 4 yrs	83 (7.6%)	19 (7.2%)	27 (8.0%)	37 (7.5%)	0.055	37 (7.5%)	46 (7.7%)	0.01^a^
5–9 yrs	367 (33.6%)	97 (36.9%)	115 (34.0%)	155 (31.5%)		155 (31.5%)	212 (35.3%)	
10–14 yrs	552 (50.5%)	130 (49.4%)	178 (52.7%)	244 (49.6%)		244 (49.6%)	308 (51.2%)	
15–18 yrs	91 (8.3%)	17 (6.5%)	18 (5.3%)	56 (11.4%)		56 (11.4%)	35 (5.8%)	
*Gender (%)*								
Female	368 (33.7%)	94 (35.7%)	110 (32.5%)	164 (33.3%)	0.697	164 (33.3%)	204 (33.9%)	0.882
Male	725 (66.3%)	169 (64.3%)	228 (67.5%)	328 (66.7%)		328 (66.7%)	397 (66.1%)	
*Insurance (%)*								
Public Insurance	511 (46.8%)	104 (39.5%)	155 (45.8%)	252 (51.2%)	0.006^a^	252 (51.2%)	259 (43.2%)	0.022^a^
Private Insurance	445 (40.7%)	112 (42.6%)	146 (43.2%)	187 (38.0%)		187 (38.0%)	258 (42.9%)	
Other^b^	137 (12.5%)	47 (17.9%)	37 (10.9%)	53 (10.8%)		53 (10.8%)	84 (13.9%)	
*Race (%)*								
White	733 (67.5%)	181 (68.7%)	225 (66.6%)	327 (67.6%)	0.895	327 (67.6%)	406 (67.6%)	0.62
Black	257 (23.7%)	63 (24.0%)	84 (24.8%)	110 (22.7%)		110 (22.7%)	147 (24.4%)	
Asian	33 (3.0%)	6 (2.3%)	9 (2.7%)	18 (3.7%)		18 (3.7%)	15 (2.5%)	
Other Race	62 (5.7%)	13 (4.9%)	20 (5.9%)	29 (6.0%)		29 (6.0%)	33 (5.5%)	
*Ethnicity (%)*								
Hispanic or Latino	130 (11.9%)	32 (12.2%)	35 (10.4%)	63 (12.8%)	0.551	63 (12.8%)	67 (11.1%)	0.447
Not Hispanic or Latino	962 (88.1%)	231 (87.8%)	303 (89.6%)	428 (87.2%)		428 (87.2%)	534 (88.9%)	

AWHUR table of patient information with regards to demographics data from years 2018 to 2020 and in relation to the COVID pandemic

^a^Satistical Significance

^b^Self Pay and Government/Military Insurance

### AWHUR injury mechanisms

**Fig. 1:**
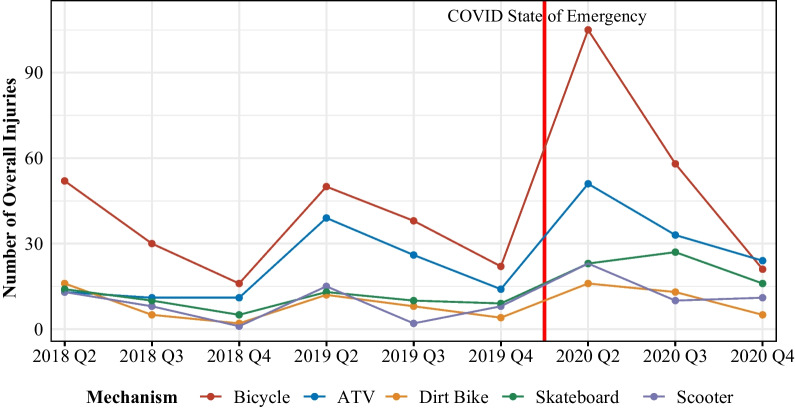
AWHUR injury mechanism trends from 2018 to 2020. This graph demonstrates the trend in top AWHUR mechanisms from 2018 to 2020 with a line indicating when the COVID-19 pandemic began along the timeline

**Fig. 2:**
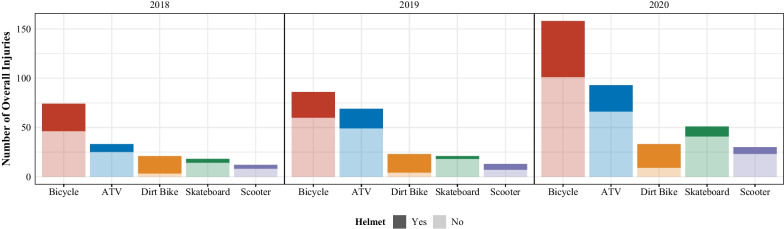
AWHUR injury mechanism and helmet status trends from 2018 to 2020. This demonstrates the number of patients who presented via the most frequent AWHUR mechanisms from 2018 to 2020. This graph also includes the breakdown of patients wearing a helmet versus not wearing a helmet for the top AWHUR mechanisms with the lighter coloration being unhelmeted patients and the darker coloration being helmeted patients

The top 5 mechanisms of injuries sustained from AWHUR were from bicycles, ATVs, skateboards, scooters, and dirt bikes accounting for 84% of all patients (Table 2 and Figs. 1 and 2). Bicycle injuries were the most common mechanism accounting for 37% of total injuries. Each year, there were more injuries secondary to these top 5 mechanisms with a large gross increase during the 2020 COVID-19 pandemic as compared to pre-pandemic years (Table 2, Figs. 1 and 2).

**Table 2:** AWHUR patient demographics, injury mechanisms, and injury severity data

	Overall	2018	2019	2020	^p^	Covid	Pre-Covid	*p*
	1093	263	338	492		492	601	
*Mechanism (%)*								
ATV	222 (20.3%)	35 (13.3%)	79 (23.4%)	108 (22.0%)	0.001^a^	108 (22.0%)	114 (19.0%)	0.003^a^
Bicycle	392 (35.9%)	98 (37.3%)	110 (32.5%)	184 (37.4%)		184 (37.4%)	208 (34.6%)	
Dirt Bike	81 (7.4%)	23 (8.7%)	24 (7.1%)	34 (6.9%)		34 (6.9%)	47 (7.8%)	
Electric Scooter	15 (1.4%)	4 (1.5%)	6 (1.8%)	5 (1.0%)		5 (1.0%)	10 (1.7%)	
Go cart	27 (2.5%)	9 (3.4%)	15 (4.4%)	3 (0.6%)		3 (0.6%)	24 (4.0%)	
Equestrian	38 (3.5%)	16 (6.1%)	11 (3.3%)	11 (2.2%)		11 (2.2%)	27 (4.5%)	
Hoverboard	34 (3.1%)	7 (2.7%)	11 (3.3%)	16 (3.3%)		16 (3.3%)	18 (3.0%)	
Roller skating	27 (2.5%)	6 (2.3%)	13 (3.8%)	8 (1.6%)		8 (1.6%)	19 (3.2%)	
Rollerblading	13 (1.2%)	3 (1.1%)	7 (2.1%)	3 (0.6%)		3 (0.6%)	10 (1.7%)	
Scooter	91 (8.3%)	22 (8.4%)	25 (7.4%)	44 (8.9%)		44 (8.9%)	47 (7.8%)	
Skateboard	127 (11.6%)	29 (11.0%)	32 (9.5%)	66 (13.4%)		66 (13.4%)	61 (10.1%)	
Other^b^	26 (2.4%)	11 (4.2%)	5 (1.5%)	10 (2.0%)		10 (2.1%)	16 (2.6%)	
*Helmet (%)*								
Helmet	309 (37.1%)	78 (41.3%)	91 (37.0%)	140 (35.2%)	0.36	140 (35.2%)	169 (38.9%)	0.305
No	524 (62.9%)	111 (58.7%)	155 (63.0%)	258 (64.8%)		258 (64.8%)	266 (61.1%)	
Unknown	260 (23.8%)	74 (28.1%)	92 (27.2%)	94 (19.1%)		94 (19.1%)	166 (27.6%)	
*Glasgow coma score (%)*								
13 to 15	1045 (95.9%)	253 (96.2%)	326 (96.4%)	466 (95.3%)	0.508	466 (95.3%)	579 (96.3%)	0.687
8 or less	31 (2.8%)	5 (1.9%)	10 (3.0%)	16 (3.3%)		16 (3.3%)	15 (2.5%)	
9 to 12	14 (1.3%)	5 (1.9%)	2 (0.6%)	7 (1.4%)		7 (1.4%)	7 (1.2%)	
*Head Injury (%)*								
Head Injury	281 (26.5%)	56 (22.3%)	85 (25.8%)	140 (29.3%)	0.119	140 (29.3%)	141 (24.3%)	0.076
None	778 (73.5%)	195 (77.7%)	245 (74.2%)	338 (70.7%)		338 (70.7%)	440 (75.7%)	
*Injury severity score (%)*								
Mild (1–8)	740 (70.0%)	186 (74.4%)	230 (69.9%)	324 (67.8%)	0.065	324 (67.8%)	416 (71.8%)	0.035^a^
Moderate (9–15)	238 (22.5%)	50 (20.0%)	70 (21.3%)	118 (24.7%)		118 (24.7%)	120 (20.7%)	
Severe (16–24)	53 (5.0%)	13 (5.2%)	21 (6.4%)	19 (4.0%)		19 (4.0%)	34 (5.9%)	
Very severe (> 24)	26 (2.5%)	1 (0.4%)	8 (2.4%)	17 (3.6%)		17 (3.6%)	9 (1.6%)	
*Neurosurgery consult (%)*								
No	935 (85.5%)	246 (93.5%)	284 (84.0%)	405 (82.3%)	< 0.001^a^	405 (82.3%)	530 (88.2%)	0.008^a^
Yes	158 (14.5%)	17 (6.5%)	54 (16.0%)	87 (17.7%)		87 (17.7%)	71 (11.8%)	

^a^Staistical Significance

^b^Other includes: Tricycle, Segway, Powerwheel, Motorized Car, Motorcycle, Ice Skating, Moped

AWHUR table of patient information with regard to injury mechanisms and injury severity-related data from years 2018 to 2020 and in relation to the COVID pandemic

### Helmet use

Helmet use was documented for 833 (76%) of patients; of these, 62.9% of patients were unhelmeted at the time of injury (Table 2). During the pandemic year, 258 patients presented unhelmeted at a rate of 64.8% compared to a rate of 61.1% for the pre-pandemic period (*p* = 0.305) (Table 2). By age groups, the number of children presenting unhelmeted were 65% of children less than 4 years old, 61% of children 5 to 9 years old, 62% of children 10 to 14 years old, and 71% of children 15–18 years old (*p* = 0.53). Over the 3-year period, publicly insured patients presented unhelmeted approximately 66% of the time while privately insured children presented unhelmeted 44% of the time (*p* < 0.001). Over the 3 years, dirt bike injuries presented with a helmeted rate of 79.2%.

### Head injuries and injury severity

Over the entire study period, 96% of patients presented with a GCS of 13 to 15 (Table 3). The proportion of children presenting with a GCS of 8 or less was 2.5% pre-pandemic compared to 3.3% during the pandemic (*p* = 0.50) (Table 2). Of patients with a head injury diagnosis via ICD coding, 19% of helmeted patients had a head injury compared to 41% of unhelmeted patients (*p* < 0.001) (Table 3). Unhelmeted patients were more likely to have a GCS < 13 (7.1%) versus helmeted patients (0.9%) (*p* < 0.001). Additionally, 8% of patients who were wearing a helmet required an emergent neurosurgical consultation compared to 24% of patients who presented without a helmet (Table 3).

**Table 3:** AWHUR helmeted injury mechanisms and injury severity data

	Overall	Helmet—Yes	Helmet—No	*p*
	1093	309	524	
*Mechanism (%)*				
ATV	222 (20.3%)	55 (17.8%)	140 (26.7%)	< 0.001^a^
Bicycle	392 (35.9%)	111 (35.9%)	207 (39.5%)	
Dirt Bike	81 (7.4%)	61 (19.7%)	16 (3.1%)	
Electric Scooter	15 (1.4%)	6 (1.9%)	7 (1.3%)	
Go cart	27 (2.5%)	10 (3.2%)	10 (1.9%)	
Equestrian	38 (3.5%)	18 (5.8%)	9 (1.7%)	
Hoverboard	34 (3.1%)	4 (1.3%)	9 (1.7%)	
Roller skating	27 (2.5%)	1 (0.3%)	3 (0.6%)	
Rollerblading	13 (1.2%)	1 (0.3%)	1 (0.2%)	
Scooter	91 (8.3%)	17 (5.5%)	38 (7.3%)	
Skateboard	127 (11.6%)	17 (5.5%)	73 (13.9%)	
Other^b^	26 (2.4%)	8 (2.6%)	11 (2.2%)	
*GCS (%)*				
13 to 15	1045 (95.9%)	306 (99.0%)	486 (92.9%)	< 0.001^a^
8 or less	31 (2.8%)	2 (0.6%)	25 (4.8%)	
9 to 12	14 (1.3%)	1 (0.3%)	12 (2.3%)	
Head Injury (%)				
Yes	281 (26.5%)	56 (18.8%)	209 (41.3%)	< 0.001^a^
No	778 (73.5%)	242 (81.2%)	297 (58.7%)	
*Injury severity score (%)*				
Mild (1–8)	740 (70.0%)	210 (70.7%)	312 (61.8%)	0.028^a^
Moderate (9–15)	238 (22.5%)	70 (23.6%)	139 (27.5%)	
Severe (16–24)	53 (5.0%)	13 (4.4%)	36 (7.1%)	
Very Severe (> 24)	26 (2.5%)	4 (1.3%)	18 (3.6%)	
*Neurosurgery consult (%)*				
No	935 (85.5%)	283 (91.6%)	401 (76.5%)	< 0.001^a^
Yes	158 (14.5%)	26 (8.4%)	123 (23.5%)	

AWHUR table of patient information with regards to helmet usage, and injury mechanisms and injury severity from 2018 to 2020

^a^ Statistical Significance

^b^ Other: Tricycle, Segway, Powerwheel, Motorized Car^−^ Motorcycle, Ice Skating, Moped

The overall ISS of patients during COVID-19 with a very severe score (> 25) was 3.6% compared to pre-pandemic average of 1.6%. Breaking down the ISS by helmet status over the 3 years, 1.3% of helmeted patients had a score > 25 compared to 3.6% of unhelmeted patients (*p* = 0.035) (Table 3). Roughly, 30% of helmeted patients had a score > 9 consistent with mild injury compared to roughly 38% of unhelmeted patients with a comparable ISS (*p* = 0.02).

## Discussion

This study discovered a statistically significant increase in overall injuries sustained from AWHUR from 1.85 injuries per 10,000 children in 2018 up to 3.43 injuries per 10,000 in 2020 in a large metropolitan region in the context of the COVID-19 pandemic. For the five most common injury mechanisms, there was a statistically significant increase in the proportion of these injuries occurring during COVID-19 except for dirt bikes. These results align with other studies finding an increase in general non-motorized vehicle injuries during the first year of the pandemic compared to prior years in pediatric trauma centers (Bessoff et al. 2021; Schroeder and Cowhig 2020). Although the change of injury occurrences varies in the literature, the increase in bicycle injury presentations throughout the initial stages of the pandemic aligned with previous studies, but this study did not align with other publications reporting a decrease in ATV injuries (Shack et al. 2022; Sanford et al. 2021).

Upon the evaluation of AWHUR injuries demographics, it was determined that almost 85% of the patients injured were school aged children between 5 and 14 years old and thus had likely experienced significant school disruptions from the COVID-19 pandemic. Multiple studies have documented the pandemic’s disruption of child care and family routines leading to the potential for these injuries (Sanford et al. 2021). Notably, we also found an increase in the proportion of publicly insured children sustaining injuries from AWHUR during the pandemic compared to previous years. This aligns with research that families with less economic resources experienced more instability and stress from the COVID-19 pandemic (McGeehan et al. 2004).

This study documented no statistically significant change in helmet usage, which is new data that has not been previously published. However, even with stable helmet use rates, given the increasing numbers of children engaging in AWHUR, there was an increase in the gross number of head injuries and patients presenting without a helmet at the time of injury. In evaluating these head injuries and helmet usage, our data align with previous research finding that helmets reduce the severity of TBI (Lee et al. 2022).

Similar to the existing literature, this study found that 66% of publicly insured children presented unhelmeted at the time of their injury. This finding is notable given that the proportion of publicly insured patients injured from AWHUR increased during the pandemic. Given that publicly insured patients are more likely to present unhelmeted and this population may have had more significant impacts from pandemic-induced societal disruptions as documented in other studies, this is a population that may have the most benefit from targeted helmet interventions (McGeehan et al. 2004; Ali et al. 2020; Gulack et al. 2015).

Although most patients presented with a GCS of 13 to 15 during the 3-year time period (96%), there was an alarming trend toward an increase in children with a GCS of less than 8 from 2.5% pre-pandemic to 3.3% during the pandemic, although this was not statistically significant. Additionally, finding that approximately 7% of children presenting unhelmeted had a GCS of 12 or less compared to 0.9% of helmeted patients again aligns with our knowledge that helmets can reduce severe brain injury (Lee et al. 2022; Gulack et al. 2015). While a wide variety of factors can influence neurosurgical consultation and it may be influenced by local practice, it is also important to note the increase in neurosurgical consultation from 6.5% in 2018 to 17.7% of patients in 2020. This study also found an increase in neurosurgical consultation in the unhelmeted population, as only 8% of helmeted patients required a neurosurgical consultation versus 3 times that occurrence in our unhelmeted population at 24%. Similarly, with the increase in our unhelmeted publicly insured population, there is concern that these children may account for these services, and additional research is needed to address this area. In addition to the above head injury findings during the pandemic, similar to other major children’s hospitals in the USA, this study discovered an increase in the ISS of our patients (Shack et al. 2022). It is alarming to see the overall ISS of injured patients during COVID-19 with a very severe score of greater than 25 was 3.6% compared to pre-pandemic average of 1.6% during 2018 and 2019.

### Limitations

Our study included patients who met the standardized national criteria to be included in the trauma registry, but it did not include all patients who presented after AWHUR injuries to our emergency department. The trauma registry is a national data base that has specific criteria for inclusion, so if a patient’s injury did not align with the national guidelines, it was not included in the registry for our review. As with any electronic health record, provider documentation was another limiting factor, as provider documentation of the injury event, injury characteristics, and ICD diagnosis coding were dependent on physician charting. This allows for physician variability in documentation impacting study results due to under or over documentation. As mentioned above, we were only able to determine helmet status in roughly 76% of children in our data analysis. Additionally, we were unable to determine if there were changes in pediatric transport practices during the pandemic, such as changes in emergency medicine service transport patterns or caregivers opting to utilize different healthcare settings for injuries sustained by children or whether to even seek out medical care at all. Patients potentially not accounted for include those that presented to local emergency departments without referral to our facility, urgent care locations, death prior to the emergency department arrival, or possibly diversion due to saturation of available hospital resources, although typically one of the pediatric trauma centers is accepting pediatric trauma patients.

## Conclusions

This study found an increase in patients presenting with injuries sustained while engaged in AWHUR during the COVID-19 pandemic. Concerningly, while helmet usage remained unaltered during the pandemic compared to previous years, there was a trend toward increased associated head injuries, injury severity scores, and subsequent neurosurgical consultations with lower GCS in the setting of increased pediatric injuries. In sum, children engaged in more recreational activities where helmets should be used; but without significant public health focus on helmet utilization, helmet usage rates remained stable. Thus, there remains a critical need to improve helmet usage with the increasing number of injuries. This should be especially targeted toward high-risk populations, such as those with public insurance as identified in this study. Further analysis is needed into the communities impacted by AWHUR-related injuries, especially after the initiation of the COVID-19 pandemic. Public health campaigns addressing safe measures, including helmets, during common childhood activities are of increasing importance.

## Methods

### Study overview

A cross-sectional retrospective chart review was conducted of the trauma registry at a Level 1 and Level 2 pediatric trauma center within a single pediatric healthcare system serving a major metropolitan area. The combined average patients seen in these two EDs was approximately 180,000 visits annually. Injuries sustained, while children participated in AWHUR were identified via the trauma registry. AWHUR were defined as activities involving bicycling, scootering (powered and un-powered, including hoverboards), off-road-vehicles (ATVs, go-karts, etc.), skiing, snowboarding, ice skating, roller skating, rollerblading, skate boarding, and equestrian sports.

### Study population

Children aged 0 to 18 years old were eligible for the study if they presented to include EDs between March 14 and December 31 of 2018, 2019, and 2020, and they were included in the trauma registry. These time periods were chosen to coincide with the public health state of emergency declared by the state. The registry data are defined by the American College of Surgeons National Trauma Standard Data Set. The main patient qualifications to be included in the registry are: one or more traumatic injuries sustained within the previous 14 days of a hospital encounter; direct hospital admission or transferred from another facility via emergency medical services (EMS) or air ambulance; death in the ED; and presentation as a trauma activation requiring surgical presence per hospital guidelines.

Patients were broadly included from the above-mentioned trauma registry based on the following International Classification of Diseases (ICD)—10 codes: S00-S99, T07, T14, and T79.A1-T79.A9 with one of the injuries in the following codes: S00, S10, S20, S30, S40, S50, S60, S70, S80, and S90 (Table 4). V00-V99 ICD-10 coded patients were included based off their external mechanism, while S00-S99 provided us patients based on their presenting injury. Patients queried from the registry via ICD-10 codes with clear injury mechanisms consistent with an AWHUR were included in the patient database; an additional manual chart review was performed to determine if a patient’s presentation was consistent with an AWHUR. Exclusion criteria included patients who were older than 18 years of age, or patients who presented via an injury mechanism not included in above-mentioned AWHUR criteria. Additionally, children who were not directly participating in the AWHUR were excluded (e.g., patient struck by another child riding a bicycle).

**Table 4:** AWHUR ICD-10 codes

ICD 10 Codes	
T07	Injuries involving multiple body regions
T14	Injury of unspecified body region
T79	Certain early complications of trauma
S00-S09	Injuries to the head
S10-S19	Injuries to the neck
S20-S29	Injuries to the thorax
S30-S39	Injuries to the abdomen, lower back, lumbar spine, pelvis, and external genitals
S40-S49	Injuries to the shoulder and upper arm
S50-S59	Injuries to the elbow and forearm
S60-S69	Injuries to the wrist, hand, and fingers
S70-S79	Injuries to the hip and thigh
S80-S89	Injuries to the knee and lower leg
S90-S99	Injuries to the ankle and foot
V00-V09	Pedestrian injured in transport accident
V10-V19	Pedal cycle rider injured in transport accident
V20-V29	Motorcycle rider injured in transport accident
V30-V39	Occupant of three-wheeled motor vehicle injured in transport accident
V60-V69	Occupant of heavy transport vehicle injured in transport accident
V80-V89	Other land transport accidents
V98-V99	Other and unspecified transport accidents

Table listing the ICD codes used for obtaining our patient population from the trauma registry

Most data were abstracted from the trauma registry; individual chart review was performed if data were missing from the trauma registry. Data abstracted included demographics, insurance status, injury severity score (ISS), activity type, helmet use, presence of head injury, neurosurgical consultation, and extent of head injury. Head injuries were identified through the Glasgow Coma Scale (GCS) and ICD codes consistent with a head injury. Data were analyzed for each year to compare the same period pre-COVID and to the time frame of the statewide public health state of emergency in 2020. Children who presented with unknown helmet status after review of individual patient charts were included in mechanism analysis but were excluded from the head injury analysis in relation to helmet status.

To assess the impact of injuries within the patient community, data was pulled from the 2020 US Census to identify the pediatric population in the 17 counties that comprise the majority (97%) of ED visits at this pediatric healthcare system. This study received approval via the children’s hospital institutional review board (IRB).

### Analysis

For descriptive analyses, patient demographics and clinical characteristics were summarized using means and standard deviations or medians and interquartile ranges for continuous data and counts and percentages for categorical data. Differences in patient characteristics between pre-COVID (2018–2019) and COVID (2020) periods were assessed using chi-square tests (or Fisher’s exact tests). Trends were calculated using linear regression models with logged case counts as the dependent variable over the 3-year time period from 2018 to 2020. The number of injuries due to AWHUR were standardized by dividing our patient catchment population with US Census Data, multiplied by 10,000. The annual rate of change and corresponding to 95% confidence interval (CI) were reverse transformed for interpretation and reported. The different distribution in age, insurance status, injury, and requiring neurosurgical consultation between injuries with helmet use and injuries without helmet use was measured by chi-square tests. All statistical analyses were performed using SAS software (v9.4; SAS Institute, Cary, NC) and R (v4.0.2). Statistical significance was set at *P* < 0.05.

## Data Availability

Data and materials are available to other parties for research purposes after a data sharing agreement plan is agreed to and signed.

## Abbreviations

TBI: Traumatic brain injury
AWHUR: Activities where helmet use is recommended
ATV: All-terrain vehicle
ED: Emergency department
AAP: American Academy of Pediatrics
CDC: Centers for Disease Control
ISS: Injury severity score
GCS: Glasgow coma scale
ICD: International Classification of Diseases
